# The Impact of Socioeconomic Factors, Coverage and Access to Health on Heart Ischemic Disease Mortality in a Brazilian Southern State: A Geospatial Analysis

**DOI:** 10.5334/gh.770

**Published:** 2021-01-20

**Authors:** Amanda de Carvalho Dutra, Lincoln Luís Silva, Raíssa Bocchi Pedroso, Yolande Pokam Tchuisseu, Mariana Teixeira da Silva, Marcela Bergamini, João Felipe Hermann Costa Scheidt, Pedro Henrique Iora, Rogério do Lago Franco, Catherine Ann Staton, João Ricardo Nickenig Vissoci, Oscar Kenji Nihei, Luciano de Andrade

**Affiliations:** 1 Post-Graduation Program in Health Sciences, State University of Maringá, Maringá, Paraná, BR; 2 Post-Graduation Program in Biosciences and Physiopathology, State University of Maringá, Maringá, Paraná, BR; 3 Study Group on Digital Technologies and Geoprocessing in Health (GETS), State University of Maringá Maringá, Paraná, BR; 4 Department of Medicine, State University of Maringá, Maringá, Paraná, BR; 5 Department of Surgery, Division of Emergency Medicine, Duke University Medical Center, Durham, North Carolina, US; 6 Duke Global Health Institute, Duke University, Durham, North Carolina, US; 7 Education, Letters and Health Center, State University of the West of Paraná, Foz do Iguaçu, Paraná, BR

**Keywords:** ischemic heart disease, spatial analysis, health services accessibility, epidemiology

## Abstract

**Background:**

No other disease has killed more than ischemic heart disease (IHD) for the past few years globally. Despite the advances in cardiology, the response time for starting treatment still leads patients to death because of the lack of healthcare coverage and access to referral centers.

**Objectives:**

To analyze the spatial disparities related to IHD mortality in the Parana state, Brazil.

**Methods:**

An ecological study using secondary data from Brazilian Health Informatics Department between 2013–2017 was performed to verify the IHD mortality. An spatial analysis was performed using the Global Moran and Local Indicators of Spatial Association (LISA) to verify the spatial dependency of IHD mortality. Lastly, multivariate spatial regression models were also developed using Ordinary Least Squares and Geographically Weighted Regression (GWR) to identify socioeconomic indicators (aging, income, and illiteracy rates), exam coverage (catheterization, angioplasty, and revascularization rates), and access to health (access index to cardiologists and chemical reperfusion centers) significantly correlated with IHD mortality. The chosen model was based on p < 0.05, highest adjusted R^2^ and lowest Akaike Information Criterion.

**Results:**

A total of 22,920 individuals died from IHD between 2013–2017. The spatial analysis confirmed a positive spatial autocorrelation global between IDH mortality rates (Moran’s I: 0.633, p < 0.01). The LISA analysis identified six high-high pattern clusters composed by 66 municipalities (16.5%). GWR presented the best model (Adjusted R^2^: 0.72) showing that accessibility to cardiologists and chemical reperfusion centers, and revascularization and angioplasty rates differentially affect the IHD mortality rates geographically. Aging and illiteracy rate presented positive correlation with IHD mortality rate, while income ratio presented negative correlation (p < 0.05).

**Conclusion:**

Regions of vulnerability were unveiled by the spatial analysis where sociodemographic, exam coverage and accessibility to health variables impacted differently the IHD mortality rates in Paraná state, Brazil.

**Highlights:**

- The increase in ischemic heart disease mortality rates is related to geographical disparities.
- The IHD mortality is differentially associated to socioeconomic factors, exam coverage, and access to health.
- Higher accessibility to chemical reperfusion centers did not necessarily improve patient outcomes in some regions of the state.
- Clusters of high mortality rate are placed in regions with low amount of cardiologists, income and schooling.

## Introduction

Ischemic heart disease (IHD) is projected to continue to be a major cause of death in 2030 [1]. Although the advances in cardiology, IHD has become an epidemic disease and one of the main causes of hospitalizations and morbimortality worldwide [2]. According to the World Health Organization, more than 9 million deaths from IHD were estimated in 2016 [3]. In 2015, most IHD deaths occurred in Brazil, which was the upper-middle-income country with the second-highest number of deaths [4].

It’s well known that socioeconomically disadvantaged groups are at high risk of cardiovascular diseases with poor outcome [5]. In addition, the inequalities in healthcare, major disparities in individual income and education [6], low accessibility to healthcare centers and limited health coverage offered to the patients [7] may be responsible for IHD mortality rates in Brazil.

Easy access to healthcare centers lowers the mortality and morbidity generally [8]. However, some studies have shown that the healthcare resources are unequally distributed across regions in Brazil [9]. Thus, geostatistical tools such as spatial analysis to verify the geographically variance of IHD risks could increase the optimization of healthcare resource allocations [10].

Brazil is a perfect setting for an analysis of the interaction between healthcare access, socioeconomic inequality, and outcomes. Exploring and mapping spatially the disease’s risk and mortality is determinant for its comprehension and proper healthcare organization and planning [11]. Using tools based on geographic information systems, it is possible to identify patterns associated with the sociodemographic profiles, coverage of tests, procedures and accessibility for each area, which collaborates in taking measures and modifying health policy plans [11,12].

Given this context, this study aims to analyze the spatial distribution of mortality rates due to IHD and its association with sociodemographic indicators, exam coverage, and accessibility to health in the State of Paraná, Brazil, in order to verify whether high mortality due to IHD is related to the disparities in the municipalities.

## Methods

### Study design and location

This is an ecological study using spatial analysis tools based on secondary data from IHD mortality data in Paraná State, from 2013 to 2017. For the assessment of methodological quality, we followed the recommendations from the guideline *Strengthening the Reporting of Observational Studies in Epidemiology* (STROBE) [13].

**Figure 1:**
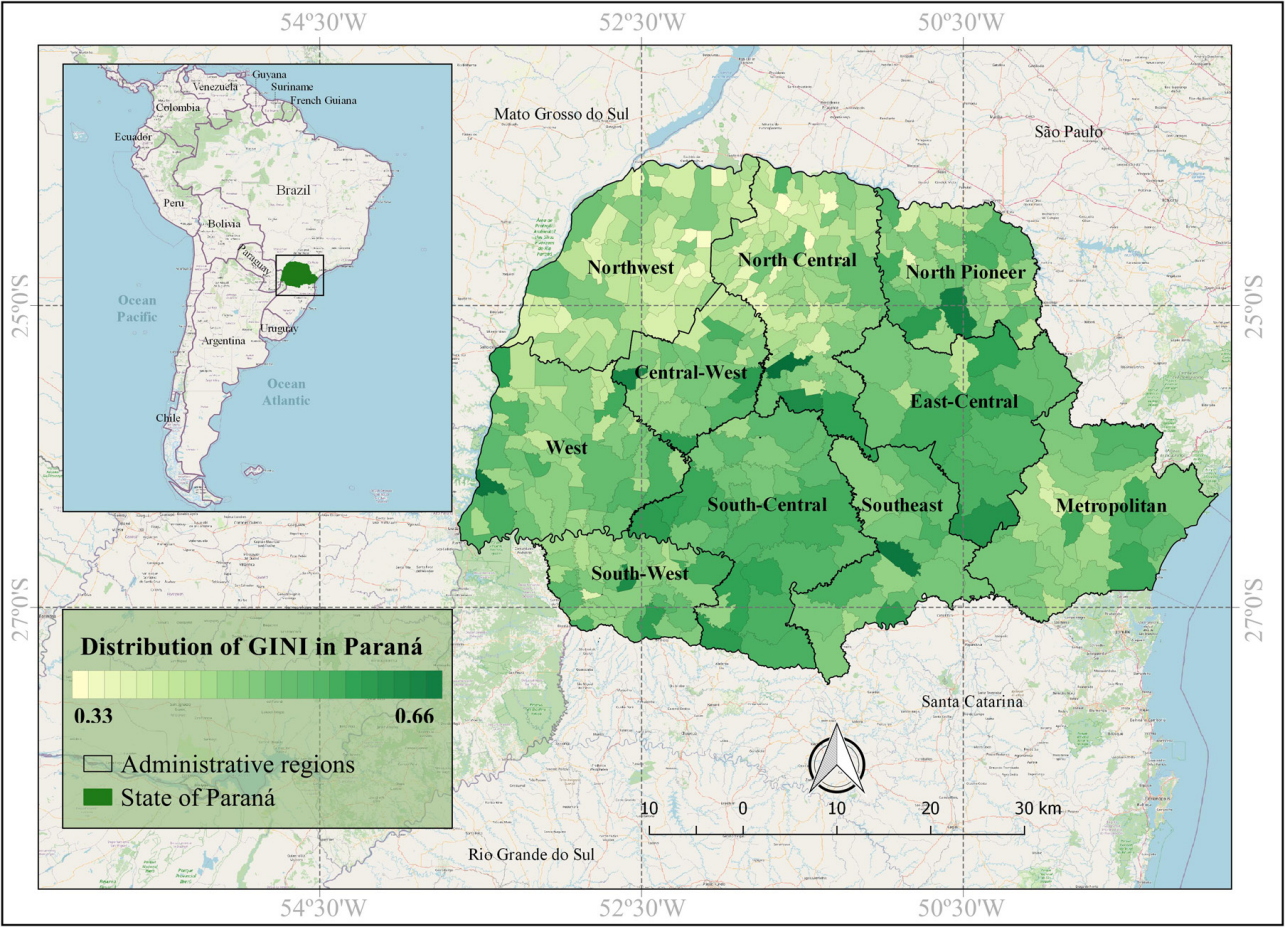
Geographic location of South America, Brazil (bigger insert) and Paraná State (green, in smaller insert) and location of Parana State’s 10 geographical mesoregions and indication of it’s municipalities according to GINI Index variation, Brazil (2019).

According to Brazilian Institute of Geography and Statistics data (2015), Paraná is located in the southern region of Brazil, occupying an area of 199,880 km^2^, with latitude coordinates 22º30’58’’ and 26º43’00’’, and longitude coordinates 48º05’37’’ and 54º37’08’’ [14]. Paraná has a total of 10,439,601 inhabitants, with the majority living in urban area (85.3%), and being the sixth most populous state in Brazil (5.5% of the total population) [14,15]. This state is bordered by the countries Argentina and Paraguay and the Brazilian states of São Paulo, Mato Grosso do Sul, and Santa Catarina. Paraná is divided by 10 geographical mesoregions, composed of 399 municipalities. The state has a GINI Index of 0.60, with values, diversified by municipality, ranging from 0.33 to 0.66 (Figure 1) [16].

## Data Information

### IHD mortality data

In the Brazilian Mortality Information System (SIM), all deaths from IHD aged between 30 and 79 years old registered with the ICD-10 (I20.0 to I25.9) in the years 2013 to 2017 [5,17] were collected. The population data by city, also aged between 30 and 79 years old, were obtained from Brasilian Institute of Geography and Statistics (IBGE) [14]. Thus, we calculated the age adjusted specific IHD mortality rate per municipality, according to the American Heart Association Guidelines [18].

### Study variables and source

In order to verify the association between IHD mortality rate and exam coverage indicators, sociodemographic variables, and accessibility to health, this study evaluated the following variables: aging rate, income, illiteracy rate, proportion of family health program coverage, catheterization rate, scintigraphy rate, angioplasty rate, chemical reperfusion rate, echocardiography rate, revascularization rate, exercise test rate, and accessibility to cardiologists, chemical reperfusion centers, and mechanical reperfusion centers [19]. These variables were selected to represent characteristics that may affect IHD mortality rates [20]. The variables of race, ethnicity, and gender were analyzed in the study but excluded because they did not have significance in multivariate regression and increased multicollinearity.

The health coverage data were obtained from the Brazilian National Register of Health Facilities (CNES) [21], while the indicator variables to create the examination rates were obtained from the Brazilian Hospital Information System (SIH) by the Microdatasus package, using the R studio software [22].

### Cartographic bases

Cartographic base data for all municipalities in Paraná State in 2017 was obtained from the Paraná Land, Cartography and Geology Institute – ITCG (www.pr.gov.br/itcg) [23].

### Data availability

All data are publicly and freely available from the Brazilian Health System Informatics Department (DATASUS). Table 1 lists all datasets accessed and utilized in this study.

**Table 1:** Data sources used for the analysis of this study.

Source	Variables	Period	Link

DATASUS – Mortality Information System (SIM)	Cause of death coded by ICD-10: I20, I21, I22, I23, I24, I25 Deaths in people between 30 and 79 years-old	2013–2017	https://datasus.saude.gov.br/
CNES National Register of Health Facilities	Geolocation of chemical reperfusion centers Geolocation of mechanical reperfusion centers Geolocation of the number of cardiologists	2013–2017	http://cnes.datasus.gov.br/
ITCG Paraná Land, Cartography and Geology Institute	Shapefile of the State and municipalities of Paraná	2015	http://www.itcg.pr.gov.br/modules/faq/category.php?categoryid=8
IBGE Brazilian Institute of Geography and Statistics	Population	2013–2017	https://www.ibge.gov.br/
PNUD United Nations Development Program	Aging rate Income ratio Illiteracy GINI	2010	http://www.atlasbrasil.org.br/2013/pt/download/
Software R (microdatasus) DATASUS – Hospital information system – SIH	Number of catheterization Number of scintigraphy Number of angioplasty Number of chemical reperfusions Number of echocardiography Number of revascularization Number of exercise test	2013–2017	https://www.scielo.br/scielo.php?script=sci_arttext&pid=S0102-311X2019001104001 https://datasus.saude.gov.br/
PMAQ Portal of the Secretariat of Primary Health Care	Proportion of family health program	2015	https://aps.saude.gov.br/ape/pmaq

## Accessibility

### Accessibility to cardiologists’ office and referral centers of chemical reperfusion and mechanical reperfusion

Two Step Floating Catchment Area (2SFCA) on ArcGIS® software (ESRI Company, USA, 2020) was used to create three accessibility indexes in order to verify the geographical accessibility (availability and proximity) [24], to cardiologists, chemical reperfusion centers and mechanical reperfusion centers. In the first step, the capacity of each center was calculated using the total number of cardiologists and service capacity available in referral centers chemical and mechanical, inside a buffer with 60 km radius surrounding each cardiologists’ office and the referral centers were created to verify the population potentially coverage [25]. For the access time, the distance of 60 km was evaluated, considering that the patient must reach the reperfusion centers within the recommended time of 60 minutes. In the first step, the capacity of each center is determined, while the second step consists of summing up the capacities within the buffer taking into account the overlapped health service area. Thus, the accessibility is the available amount of cardiologists and referral centers chemical and mechanical per local population added up within 60 km of each health service [25,26,27]. The result is an accessibility index to cardiologists, chemical reperfusion referral centers and mechanical reperfusion centers for each municipality. A higher accessibility index indicates more access to these resources for a given municipality.

## Geospatial Analysis

### Spatial inference

Spatial analysis was applied to determine the existence of significant Global Spatial Autocorrelation (Moran’s I) and Local Index of Spatial Autocorrelation (LISA) to verify the influence of spatiality on IHD mortality rate using GeoDa software version 1.12.0 [28].

Moran’s I only verifies the presence of spatiality globally, meaning that local patterns of spatial association may be hidden [29]. In order to avoid this, LISA was applied to identify the significant formation of clusters locally [30], categorized as: high-high (HH) clusters, in which they are a set of municipalities with high IHD mortality rates surrounded by other municipalities with high IHD mortality rates. On the other hand, low-low clusters (LL) are groups of municipalities with low IHD mortality rates surrounded by municipalities with low IHD mortality rates. The global and local spatial autocorrelation coefficients were considered significant when p < 0.05 [28].

### Spatial regression

To identify which sociodemographic, health coverage, procedures and health access indicators had the greatest geospatial impact on the spatial distribution of IHD mortality rate, we performed a multivariate spatial regression analysis using Ordinary Least Squares (OLS) and Geographically Weighted Regression (GWR). The OLS regression does not take spatial dependence into the analysis [31]. On the other hand, GWR considers spatial dependency by performing several separated regressions, identifying significant geographic clusters within the studied area [32]. Additionally, GWR produces an estimate for the association between IHD mortality rate and its predictor variables, from analysis of the local spatial variability for each municipality.

The selection of variables for the final model was based on the criteria of low multicollinearity, with a result of 29.7 (Conditional number test – Geoda), preventively showing that multicollinearity does not influence the regression results [31]. Thus, initially, each significant variable in the global model (OLS) were tested in the local model (GWR), allowing to verify the presence of spatial non-stationarity, and that the correlations of the regression variables vary locally in relation to space, as indicated by the chloroplastic maps, indicating the differential spatial impact of each variable on IHD mortality rates.

The chosen model between OLS and GWR was the one that provided the best fit based on the p < 0.05, highest adjusted R^2^, lowest Akaike Information Criterion (AIC) and Moran’s I residuals. GeoDa software was utilized to generate the OLS model; R Studio software was used to execute the GWR model and QGIS software, version 2.14.9, was used to generate choropleth maps.

### Ethical aspects

This study did not require ethical approval and consent form, since we used data from secondary sources in publicly available, government and online databases (https://datasus.saude.gov.br/informacoes-de-saude-tabnet/). Nevertheless, this study followed the Brazilian National Guidelines for research with human beings, according to Resolution 510/2016 of the National Health Council.

## Results

During the analyzed period, 22,920 deaths due to IHD occurred mainly among men (64.5%), aging between 70 and 79 years (36.7%), white (73.2%), married (49.1%), and with up to seven years of schooling (57.6%). Most deaths occurred in the hospital (49.5%), followed by deaths at home (30.2%).

**Figure 2:**
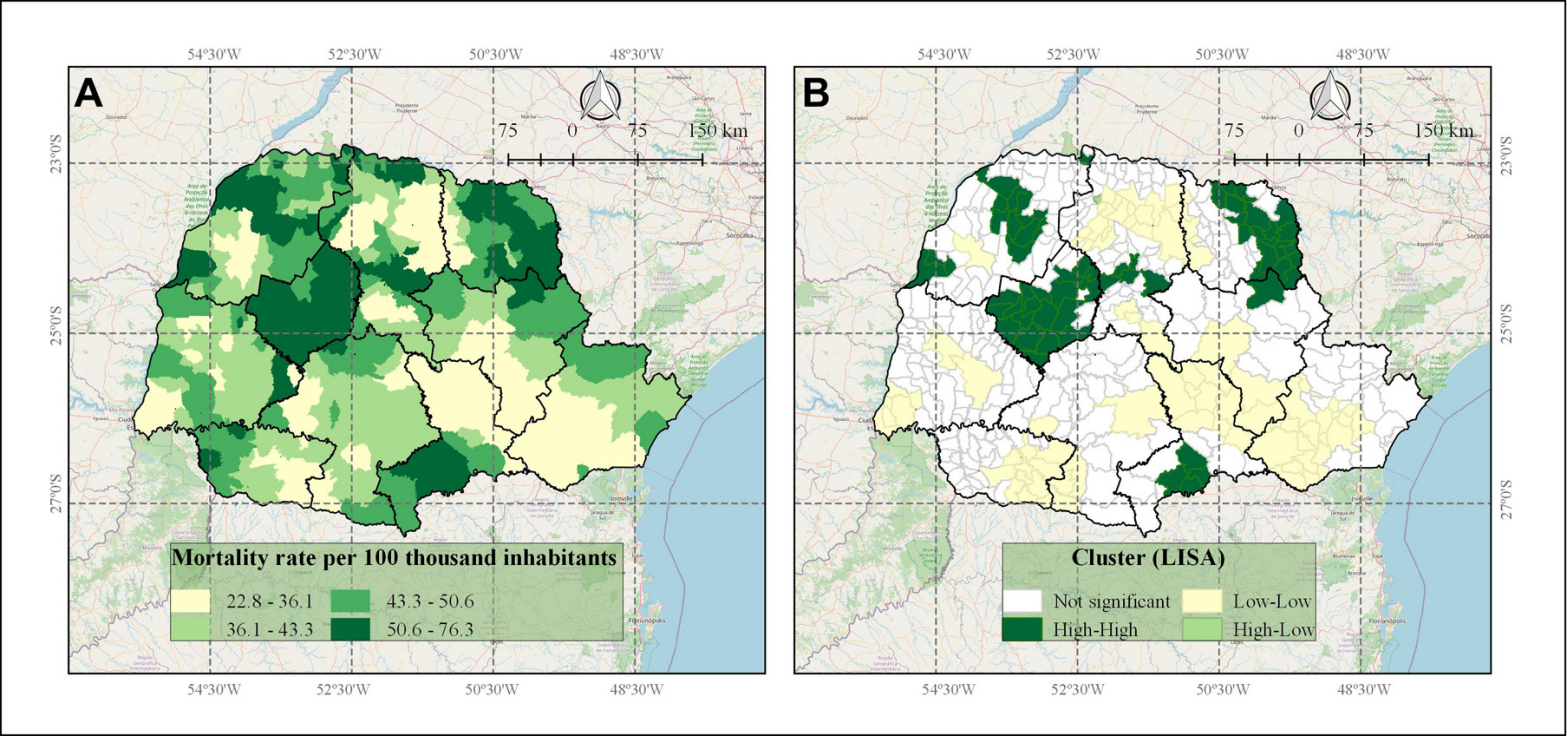
Spatial distribution of IHD mortality rates in the 399 municipalities of Parana State, Brazil, and its clusters. A) IHD mortality rates by adjusted population/100 thousand inhabitants in Parana State, 2013 to 2017; B) Local Indicators of Spatial Association (LISA) analysis indicating clusters according to high and low IHD mortality rates distribution patterns.

Regarding spatial patterns of death distribution from IHD in the 399 cities in Parana State, on average 44.0/100,000 (+/– 10.6; Standard Deviation) inhabitants died per year from IHD. The 399 cities presented IHD mortality rates between 22.8 and 76.3 per 100,000 inhabitants, these cities being located at high rates (50.6 to 76.3 per 100,000 inhabitants) mainly in Central-West, NorthWest, North Pioneer, and Southeast regions of the Paraná state (Figure 2A).

Global Moran Univariate Analysis indicated the existence of a positive spatial autocorrelation (Moran’s I = 0.633, p < 0.01), demonstrating that municipalities with high mortality rates by IHD tend to be surrounded by neighboring towns with similar high rates.

According to Figure 2B, the LISA analysis detected six high-high (HH) pattern clusters, indicating cities with high IHD mortality rates close to neighbors also showing high IHD mortality rates, covering 16.5% of the municipalities in the state. It was also observed in six low-high (LH) pattern clusters, indicating cities with low IHD mortality rates with neighbors with high IHD mortality rates. Only one high-low cluster (HL) was identified in the North Central of Paraná, indicating cities with high IHD mortality rates with neighbors with low IHD mortality rates. Considering the two largest HH clusters, one included municipalities located in the Central-West and North Central, and the other included municipalities located in the North Pioneer and East-Central of the state.

The spatial regression was performed and from the initial set of 14 variables, 8 remained significant. Table 2 presents the results of the multivariate spatial regression analysis to identify variables that were correlated with municipalities’ IHD mortality rates. According to global OLS results, the following variables were removed (p > 0.05): accessibility to chemical reperfusion centers, proportion of family health program coverage, scintigraphy rate, chemical reperfusion rate, echocardiography rate, and ergometric (exercise) test rate (Table 2). In addition, according to global OLS results, the variables that remained in the modeling process (p < 0.05) were: accessibility to cardiologists, accessibility to hospitals with chemical reperfusion, aging rate, income ratio, illiteracy, catheterization rate, angioplasty rate, and revascularization rate (Table 2).

**Table 2:** Results of global OLS and local GWR multivariate analysis considering the Parana State municipalities’ IHD mortality rates as dependent variables.

Variables	Global OLS coefficient					Local coefficientof GWR				

	Est	Std Error	T value	VIF	Pr(>|t|)	Min	IstQu	Median	3rd	Max

**Access index of Cardiologists**	–3.70	1.31	–2.83	4.17	< 0.01**	–2.59	–9.83	–4.73	–6.45	1.34
**Access of chemical reperfusion centers**	3.51	9.20	3.82	1.18	< 0.01**	–7.13	2.08	3.32	5.54	1.21
**Access of mechanical reperfusion centers**	–2.46	2.58	–0.09	3.63	0.92	–6.00	–5.86	7.20	1.33	3.37
**Aging rate**	1.02	0.28	3.64	1.42	< 0.01**	–4.31	7.07	1.03	1.34	2.22
**Income ratio**	–1.88	0.07	–2.64	1.17	< 0.01**	–1.05	–3.05	–1.89	–4.36	–6.16
**Illiteracy**	0.41	0.14	2.91	1.48	< 0.01**	–8.20	2.37	4.56	7.30	2.02
**Proportion of Family health program coverage**	–5.12	0.03	–0.19	1.29	0.85	–9.63	–3.75	1.03	4.49	1.19
**Catheterization rate**	–2.14	0.53	4.01	1.16	< 0.01**	–6.78	–2.44	–1.72	–9.86	2.08
**Scintigraphy rate**	0.28	0.22	1.28	3.56	0.20	–1.45	–4.55	9.90	6.45	1.47
**Angioplasty rate**	–2.61	1.25	–2.10	1.77	0.04*	–1.69	–6.87	–4.63	–2.24	1.66
**Chemical reperfusion rate**	–1.51	5.85	–0.26	1.13	0.80	–3.89	–7.89	1.01	9.41	5.18
**Echocardiography rate**	–1.72	1.34	–1.28	3.62	0.20	–8.63	–3.82	–1.17	2.96	9.72
**Revascularization rate**	2.71	1.19	2.28	1.79	0.02*	–8.72	–1.57	2.72	5.92	1.81
**Exercisetest rate**	0.004	0.10	0.05	2.15	0.96	–6.70	–1.29	3.37	2.42	6.37

**Intercepto**	**37.86**	**3.73**	**10.15**	–	< 0.01**	**2.07**	**3.32**	**3.62**	**4.11**	**5.09**
**Moran’s I Residuals**	**0.05**	–	–	–	–	**0.00**	–	–	–	–
**Moran’s p Residuals**	**0.00**	–	–	–	–	**0.08**	–	–	–	–
**R2 adjusted**	**0.28**	–	–	–	–	**0.72**	–	–	–	–
**AIC**	**2896.85**	–	–	–	–	**2639.51**	–	–	–	–

P valuesignificance: *<0.05; **< 0.01.

As indicated in Table 2, the GWR multivariate model, considering the significant variables, showed a significant improvement, presenting better AIC, Moran’s I residuals and adjusted R^2^ when compared with the OLS model, indicating that these variables correlate differently with IHD mortality rates in the geographical level.

**Figure 3:**
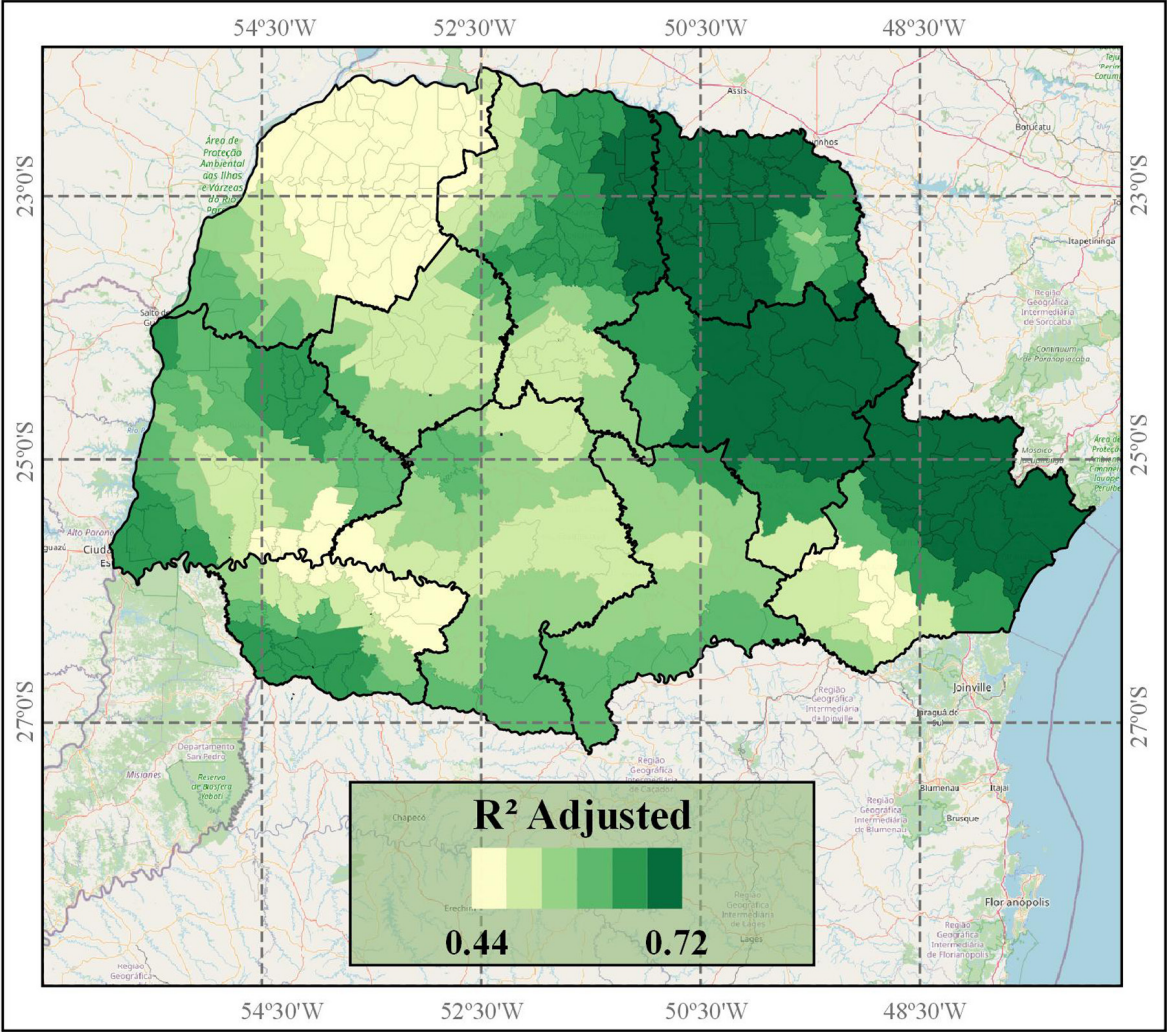
Spatial distribution of local GWR adjusted R^2^ values according to each Parana State municipality, located in mesoregions.

The GWR model had better performance in understanding the associations between the variables and IHD mortality rate. However, the model adjustment (Adjusted R^2^) varied spatially (Figure 3). The North Central, North Pioneer, East-Central, Metropolitan and parts of the West and South-West mesoregions presented better model adjustment. The Northwest, Center-West, South-Central, and Southeast mesoregions presented lower values of GWR model adjustment, below 0.6 (Figure 3).

**Figure 4:**
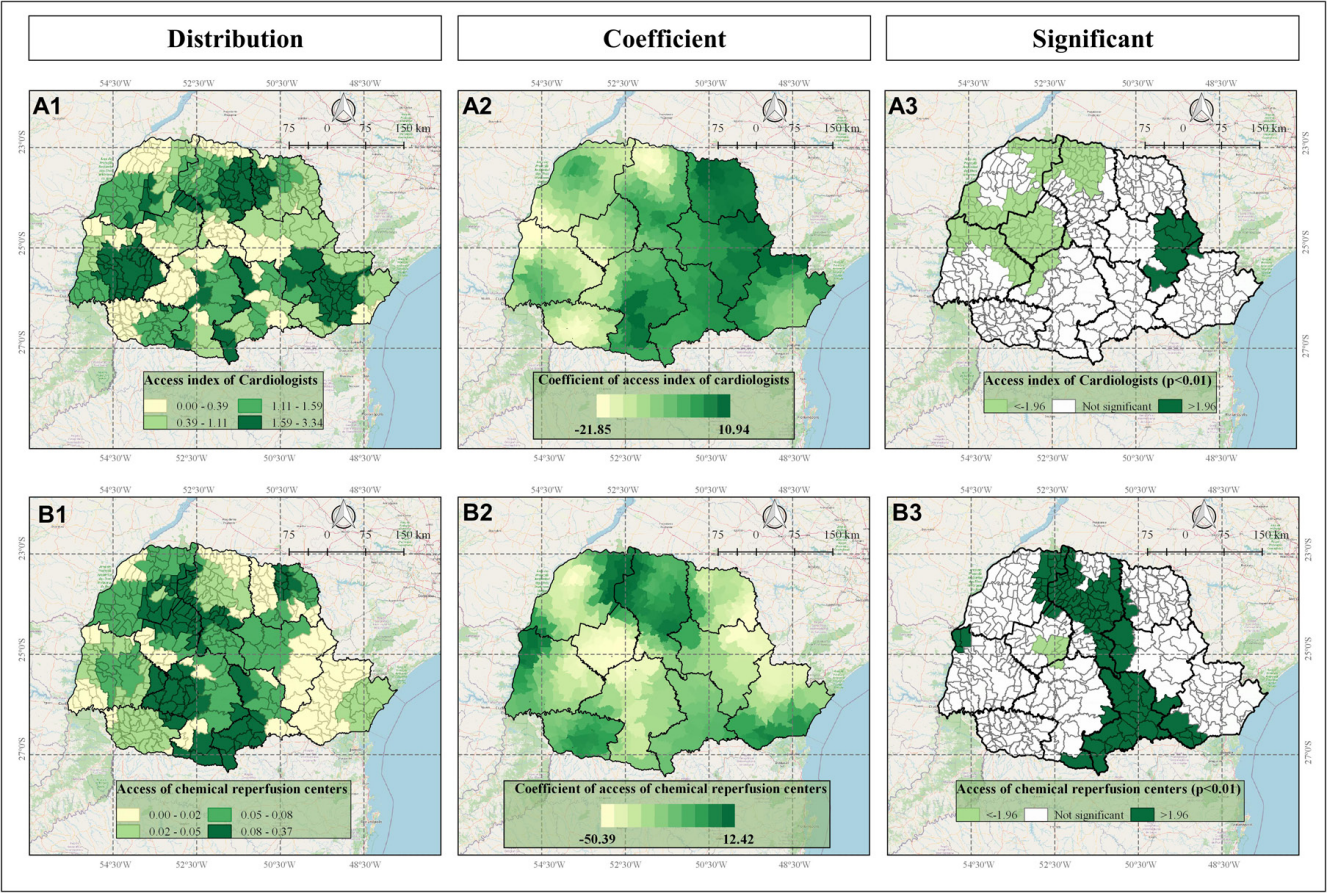
Indicators of accessibility to cardiologists (Panels A) and to chemical reperfusion centers (Panels B) in Parana state, Brazil, according to Frequency Distribution (1), values of GWR coefficients (2) and significance (3) where <– 1.96 (light green) indicates significant negative correlation and >1.96 indicates significant positive correlation (dark green).

**Figure 5:**
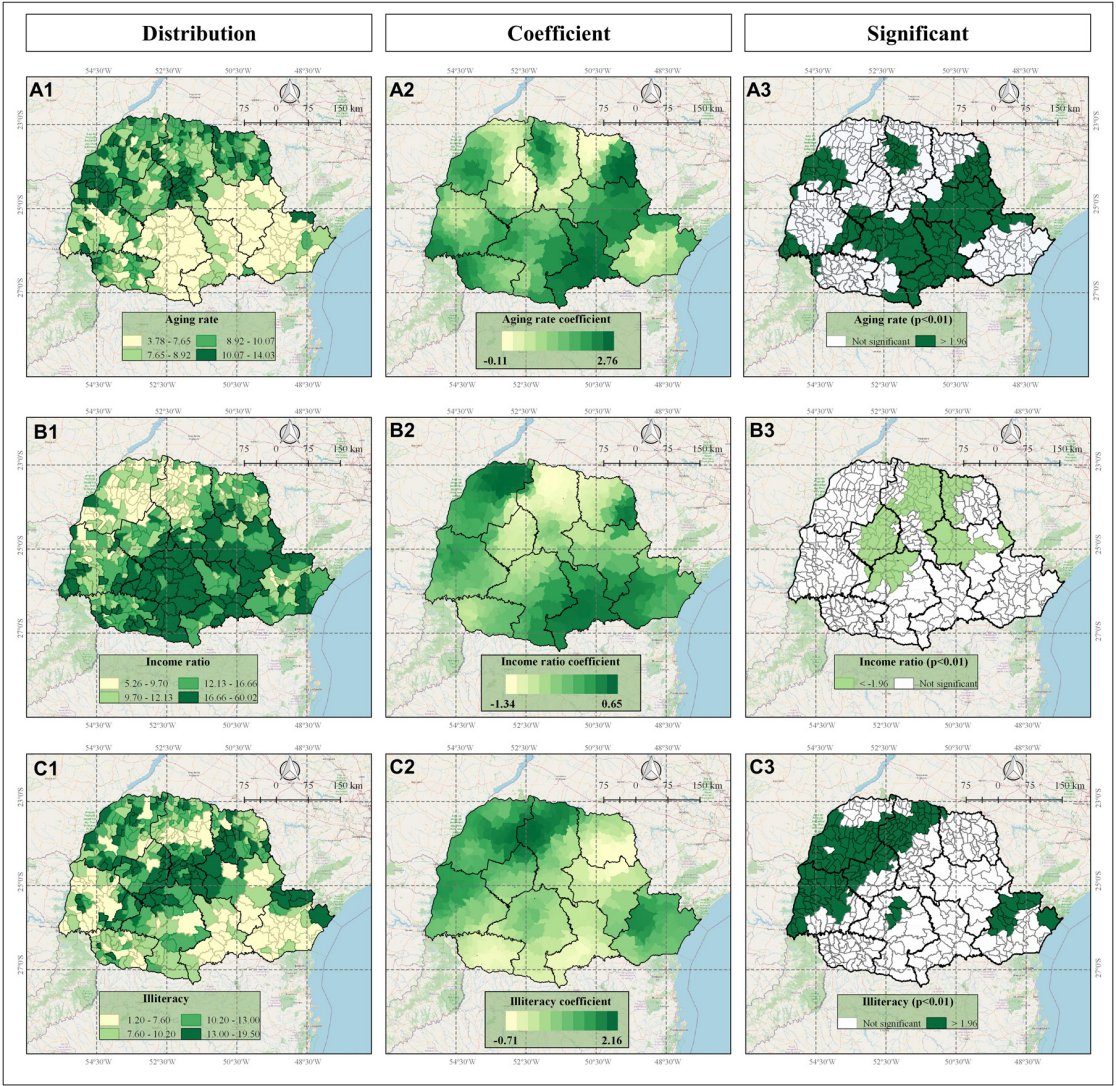
Socioeconomic indicators: aging rate (Panels A); income ratio (Panels B) and illiteracy (Panels C) in Parana state, Brazil, according to Frequency Distribution (1), values of GWR coefficients (2), and significance (3) where <–1.96 (light green) indicates significant negative correlation and >1.96 indicates significant positive correlation (dark green).

**Figure 6:**
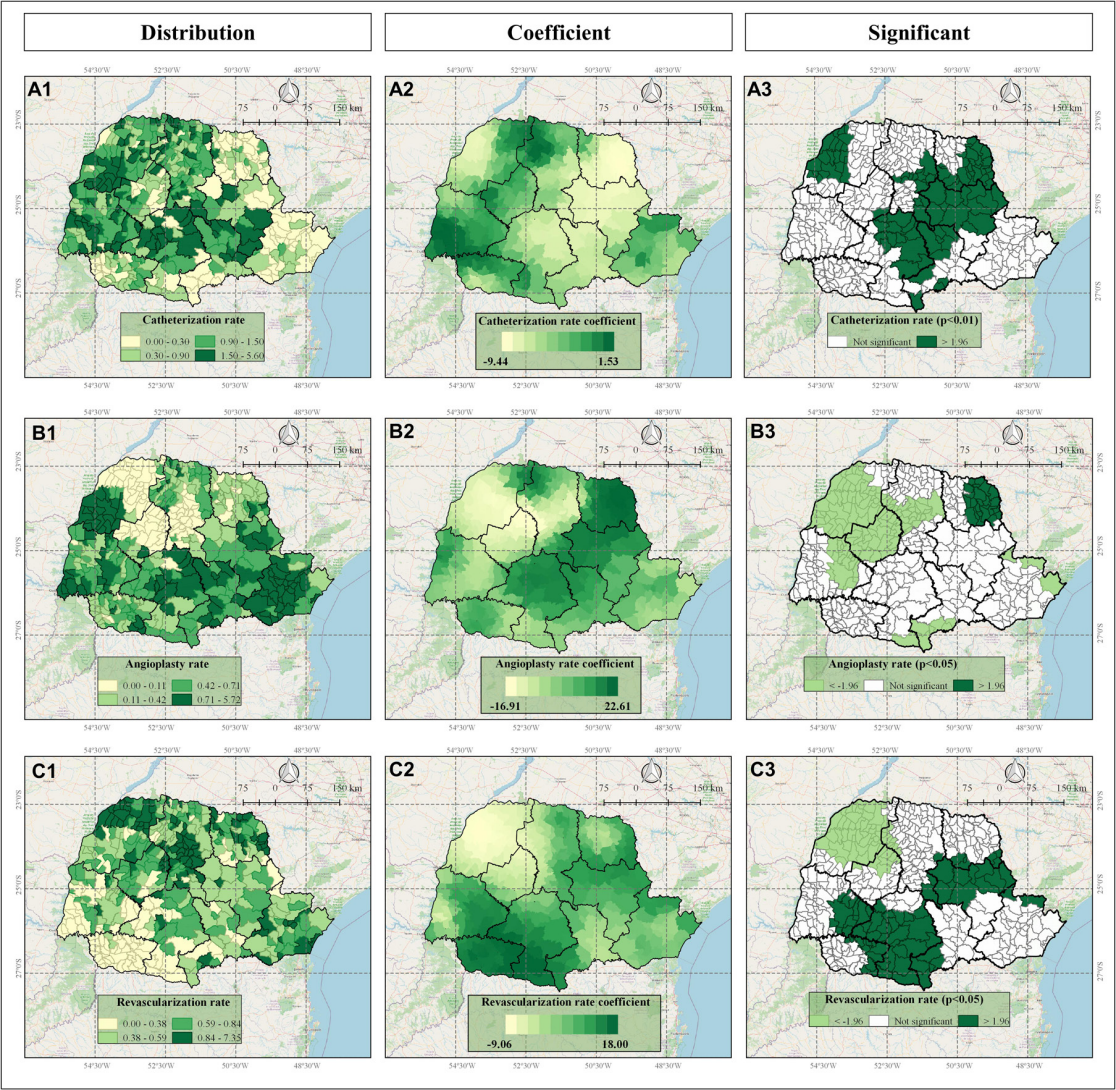
Exam coverage variables: catheterization rate (Panels A); angioplasty rate (Panels B) and Revascularization rate (Panels C) in Parana state, Brazil, according to Frequency Distribution (1), values of GWR coefficients (2), and significance (3) where <–1.96 (light green) indicates significant negative correlation and >1.96 indicates significant positive correlation (dark green).

The spatial impact of each significant variable included in the GWR model on IHD mortality rate are indicated in the Figures 4, 5, and 6.

The GWR analysis results presented the distinctive spatial impact and pattern of each variable, influencing IHD mortality rates. As indicated in Figures 4, 5, and 6, some variables presented a homogeneous influence over the municipalities, while others presented more heterogeneous spatial influence. The following variables presented negative correlation with IHD mortality rate: access to cardiologists (most on Central-West and part of Northwest, North-Central, West and Southwest mesoregions) (Figure 4 A3), access to chemical reperfusion centers (most on Central-West and few on South-Central mesoregions) (Figure 4 B3), angioplasty rate (most on Northwest, Central-West, West, and North-Central; few on South-Central, Southeast and Metropolitan mesoregions) (Figure 6 B3) and revascularization rate (most on Northwest and part of North Central and Central-West mesoregions) (Figure 6 C3). These data indicated that in these regions, higher access to cardiologists, chemical perfusion centers, and higher angioplasty rate and revascularization rate are associated with lower IHD mortality rate.

In addition, aging rate (Figure 5 A3) and illiteracy rate (Figure 5 C3) presented positive correlation, while income ratio presented negative correlation (Figure 5 B3), with IHD mortality rate, in all significant municipalities and mesoregions, indicating that municipalities’ higher aging rate and illiteracy rate and lower income ratio are associated with higher IHD mortality rates.

Concomitantly, in other mesoregions, differential spatial impact was observed concerning some of the accessibility and intervention variables and the following variables presented positive correlation with IHD mortality rate: access to cardiologists (East-Central mesoregion) (Figure 4 A3), access to chemical reperfusion centers (most on North-Central and Southeast, part of Northwest and Metropolitan, and few on West, North Pioneer and South-Central) (Figure 4 B3), catheterization rate (all significant municipalities and mesoregions) (Figure 6 A3), angioplasty rate (North Pioneer) (Figure 6 B3), and revascularization rate (most on South-Central and East-Central, part of West and Southwest and few on Southeast, North-Central, North Pioneer, and Metropolitan) (Figure 6 C3). These data indicated that in some specific municipalities and mesoregions facilitated access to healthcare and cardiological interventions did not present favorable outcomes; on the contrary, surprisingly, they were correlated with higher IHD mortality rates, suggesting that the quantity and quality of access and services in these locations are not enough and need to be improved to prevent IHD mortality. Additionally, other unidentified factors may be influencing these outcomes, as addressed in the Discussion section.

## Discussion

In this study, the spatial distribution of IHD mortality rate by municipalities was evaluated in order to verify its association with sociodemographic, exam coverage, and access to health indicators in Parana state, located in Brazil.

The GWR analysis presented the best fitted multivariate model and in the preliminary analysis showed that sex/gender and race/ethnicities were not significantly related with IHD mortality (p > 0.05) rate and were not included in the final model. This result is different from the study of Mozaffarian et al. (2015) who found that men and afro-descendants presented a higher mortality rate due to IHD in the United States [33].

The reason for such discrepancy is not known. However, the Parana state was colonized predominantly by Europeans, consisting of a white population. Thus, since the proportion of afro-descendants in Parana’s general population is very low, one possibility is that its impact on the municipalities IHD mortality rate are also very low; alternatively, it suggests that this population of men and afro-descendants may be subjected to different IHD risk factors according to Brazil’s region.

The results in our study indicated that accessibility to cardiologists, accessibility to chemical reperfusion centers, and angioplasty, catheterization, and revascularization rates presented differential spatial association with municipalities’ IHD mortality rates. The study showed that in some municipalities and mesoregions, higher access to cardiologists, chemical perfusion centers, and higher angioplasty rate and revascularization rate were associated with lower IHD mortality rates [34]. These findings are supported by the literature, because referring these patients to cardiologists care is associated with more frequent use of efficacious therapies and may improve the outcome [35].

On the contrary, in some specific municipalities and mesoregions, facilitated access to the cardiologists and chemical perfusion centers and to higher angioplasty, catheterization and revascularization rates did not present favorable outcomes, and this was correlated with higher IHD mortality rates. Although unexpected, these findings may indicate that in these specific municipalities and mesoregions, others factors may be related with patients’ poor IHD outcomes, despite the higher healthcare accessibility and higher rates of cardiological interventions. These other factors may be:

1. Patients are going to chemical reperfusion centers rather than hemodynamics. Evidence shows that reperfusion could paradoxically induce an exacerbated tissue injury and necrosis, known as reperfusion injury, which may result in poorer outcomes.
2. Chemical reperfusion centers are not well located where there is more demand of cardiologists, as shown in Figure 4. Therefore, the lack of cardiologists in these centers may affect the outcome of these patients negatively.
3. Patients have received the proper interventions; however, because some services are not well-structured or because of higher service demands (higher number of IHD cases) or a lack of professionals, the patients are not being submitted to cardiac procedures at the correct time for intervention. According to some studies, the delay in revascularization and other procedures may increase morbidity and results in increase in mortality rates [36,37,38].
4. The cardiological healthcare structure (professionals, equipments, ambulances, etc.) and services (angioplasty, catheterization, chemical reperfusion, etc.) are not homogeneously spatially distributed in Parana state and not all services are offered depending on the mesoregion, and in addition, also the procedure offering do not guarantee that professionals and cardiologist physicians are present in sufficient number.

Regarding the sociodemographic variables, aging rate and illiteracy rate presented positive correlation with IHD mortality rate, while income ratio presented negative correlation. Other studies also indicated the same correlation in high- and middle-income countries [39,40]. Lower health literacy is an invisible barrier to healthcare delivery, which has been associated with limited knowledge of health conditions, medications, poorer overall health status, higher healthcare costs, and mortality [41]. Additionally, the study by Paasche-Orlow et al. (2005) corroborates our study because they showed that lower health literacy is more prevalent among older adults and individuals with less education [42]. In addition, a similar study also analyzed income and IHD mortality rates and the mortality were higher in low middle income regions because these regions have more difficulties than high-income regions to find resources for the treatment of IHD patients [43]. Therefore, illiteracy, aging, and low income lead to poor outcome of IDH patients.

Geographic disparity in cardiovascular disease interventions is common worldwide [44]. A study in the United States of America found that < 50% of patients were transferred to hospitals for cardiac catheterization [45], increasing patients’ poor outcomes. The present study also indicated that low accessibility to healthcare and low rates of interventions are associated with higher IHD mortality rates in some mesoregions of Parana state.

In the present study, the results indicated that in some specific municipalities and mesoregions of Parana state improvement of health accessibility and exam coverage were not associated with decrease of IHD mortality rates, so future studies are needed to identify the possible other factors involved in these specific settings and scenarios.

However, the data of the present study support that the expansion of the hospitals with high complex procedures must be considered; in addition, data suggest that public health policies aiming to reduce the time of the initial cardiological treatment of IHD patients may reduce the poor outcomes of these patients and decrease the Parana state IDH mortality rates, considering the spatial differences and specificities of each mesoregion, socioeconomic factors, and healthcare accessibility scenarios.

## Limitations

There are some limitations that need to be considered in the present study such as the use of secondary data, which may present under-notifications of IHD mortality cases in Parana state. However, the data quality of the Mortality Information System obtained from the website of the Brazilian Ministry of Health has increased its credibility and has been improved. In addition, since the number of cases of IHD mortality are high the final effect of the under-notifications is possibly low.

## Conclusion

For the spatial distribution of IHD mortality rates, the association of risk indicators had an important influence on the clusters found, and it was possible to estimate the differential spatial impact of level of accessibility, assistance of health services, and socioeconomic factors on these rates in Parana state mesoregions. The study indicated the complexity of the factors associated with IDH mortality rates unveiled by the spatial analysis, showing the distinctive scenarios of intervention, indicating the vulnerable areas where public interventions may occur, which may facilitate the allocation of health resources and application of more suited prevention policies.

## Data Availability Statement

Those interested in the specific data and codes used in this study can enter the corresponding link: https://figshare.com/search?q=10.6084%2Fm9.figshare.13042649.
